# Evaluation of Stigma Toward Fatty Liver Disease

**DOI:** 10.2174/0118715303359141250420070406

**Published:** 2025-05-06

**Authors:** Yasser Fouad, Alaa M. Mostafa, Ziyan Pan, Shaymaa Nafady, Hany Samir Rasmy, Shaker Wagih Shaltout, Dalia Hassan, Mohamed Alboraie, Mohammed Eslam

**Affiliations:** 1 Department of Gastroenterology, Hepatology and Endemic Medicine, Faculty of Medicine, Minia University, Minia, Egypt;; 2 Storr Liver Centre, Westmead Institute for Medical Research, Westmead Hospital and University of Sydney, NSW, Sydney, Australia;; 3 Department of Gastroenterology, Hepatology and Infectious Diseases, Beni-Suef University, Beni- Suef, Egypt;; 4 Department of Internal Medicine, Ain Shams University, Cairo, Egypt;; 5 Department of Tropical Medicine, Port Said University, Port Said, Egypt;; 6 Department of Medicine, Alexandria University, Alexandria, Egypt;; 7 Department of Internal Medicine Institute, Al-Azhar University, Cairo, Egypt

**Keywords:** MAFLD, MASLD, fatty, alcoholic, stigma, health

## Abstract

**Background and Aims:**

A recent Delphi statement proposed an adapted term from metabolic dysfunction-associated fatty liver disease (MAFLD), metabolic dysfunction-associated steatotic liver disease (MASLD), substituting “fatty” with “steatotic”, as the former was claimed to be stigmatizing. In this cross-sectional study, we aimed to investigate this and understand stigma and awareness among fatty liver disease patients.

**Methods:**

A semi-structured interview consisting of questions was carried out with patients to get an understanding of their knowledge of fatty liver disease and their opinions on the terminology used for its diagnosis.

**Results:**

Surveys were completed by 222 fatty liver disease patients in a written questionnaire. Out of the participants, only 84 (37.8%) and 37 (16.7%) of patients believed that cirrhosis or hepatocellular carcinoma might result from fatty liver disease, while 82 (36.9%), 87 (39.2%), and 52 (23.4%) of patients thought fatty liver was connected to diabetes or cardiovascular disease. 189 individuals (85.1%) said that including the word “alcohol” in the name of a condition carries a stigma. Conversely, 177 (79.7%) of participants said that they do not consider the term “fatty” to be stigmatizing. Additionally, the degree of awareness of the disease's consequences seems very low. 159 (71.9%) and 180 (81.1%) participant expressed their lack of knowledge on how the disease can be diagnosed or treated, respectively, and 146 (65.8%) of the participants showed a desire to acquire knowledge about the impact of fatty liver disease on their well-being and health.

**Conclusion:**

A part of our concern about fatty liver disease awareness without trivialization. The use of the word “fat” in the nomenclature of fatty liver disease linked with metabolic dysfunction is not perceived to be stigmatizing. The suggested change to MASLD does not seem warranted, at least if the stigma is the main concern.

## 1 INTRODUCTION

Metabolic dysfunction-associated fatty liver disease (MAFLD) is the primary chronic liver disease that affects nearly one-third of the global population and is currently one of the leading indications for liver transplantation and hepatocellular carcinoma (HCC) in various parts of the world [1-4]

Although MAFLD is becoming more prevalent worldwide, there is a significant dearth of knowledge about the condition among healthcare professionals and the general public, particularly those who are most vulnerable to negative consequences [5], that was suggested it may be partially due to the former nomenclature used to describe this liver disease (non-alcoholic fatty liver disease). This pattern seems to have started to improve since the introduction of the MAFLD definition [6, 7]. Additionally, the diagnostic criteria of MAFLD [8-10] have been unequivocally demonstrated to have superior clinical utility in identifying hepatic and extrahepatic consequences of the disease, including among patients with co-existing other liver diseases [11].

In 2023, a Delphi consensus statement of a panel of international experts under the umbrella of three liver societies suggested another term, metabolic dysfunction-associated steatotic liver disease (MASLD), where “fatty” has been converted into its synonym “steatotic”, due to the claim that “fatty” might be stigmatizing [12]. However, evidence supporting this claim is still largely lacking. Additionally, the various terms and their related stigma are perceived differently across languages and cultures. The clinical objectives of this article are to maintain a balance between disease seriousness and stigma.

In the present study, we undertook a nationwide survey to investigate the understanding, perception, consciousness, and perceived stigma associated with the diagnostic terminology among individuals diagnosed with NAFLD/MAFLD/MASLD. Additionally, we thought to gain some insights into the knowledge of disease awareness.

## 2 METHODS

### 2.1 Study Design

In a cross-sectional study, we adopted a nationwide survey to investigate the perceived stigma associated with the diagnostic terminology among individuals diagnosed with NAFLD/MAFLD/MASLD. We aim to evaluate the impact of the word “fatty” among the participant and their knowledge of the disease sequences.

### 2.2 Participants

Two hundred and twenty-two individuals were included in this research, sought medical care at six Egyptian tertiary hospital Hepatology clinics, and were diagnosed with fatty liver disease. Inclusion criteria include all patients > 18 years old with no gender or racial considerations. Patients with significant alcohol consumption, Autoimmune liver disease, and cholestasis were excluded. The analysis only included patients who completed the survey up to the last question. All patients completed the survey without interference. Written informed consent was obtained from the participants. All patient data were included in the study regardless of their answer, and the survey results were analyzed collectively and quantitatively. This study was done in compliance with the Declaration of Helsinki, and approved by the Ethics Committee of Medical Research- Minia University.

### 2.3 Survey

A semi-structured interview consisting of 14 questions was carried out with patients to get their understanding of fatty liver disease and their level of contentment with the terminology used in the several suggested classifications of the condition. Firstly, this survey includes a concise demographic component: age, gender, educational attainment, income level, and residential status (urban or rural). The following questions must be answered by the participant without doctor interruption or interference. (Q1: “Are you ‘aware’ of ever having liver disease or have you ever been told by a doctor or other health professional that you had any kind of liver condition?”) Either yes or no. (Q2: “Have you ever heard of Non-alcoholic Fatty Liver Disease, NASH, or even the term fat in the liver?”) Either yes or no. (Q3: “How did you hear about it?”) Answers are from a health care provider, a friend/ a family member, the news or media, the internet, other sources, or none. (Q4: “Have you heard about liver cirrhosis?”) Either yes or no. (Q5: “Do you know the difference between NAFLD and NASH?”) Either yes or no. (Q6: In a score of 1-5 (1 “Not at all Concerned” to 5 “Very Concerned”), “How would you be concerned if you were diagnosed with one of these diseases? Fatty liver disease, High blood pressure, Diabetes, and Hepatitis C”). (Q7: either yes or no, “Do you know if fatty liver can cause? Cirrhosis, hepatocellular carcinoma, Diabetes, CVD, CKD, and Cancers (non-hepatic)”). (Q8: “If you were told that you are at risk for NAFLD, do you want to learn about how it might affect your health?”) Either yes or no. (Q9: “How would you like to receive health information?”) Answers are directly from a doctor or nurse, written materials, or reordered materials. (Q10: “Do you have an idea how fatty liver can be diagnosed?”) Either yes or no. (Q11: “If a doctor told you that you need a liver biopsy to evaluate fatty liver, would you accept doing it?”) Either yes or no. (Q12: “Do you have an idea how fatty liver can be treated?”) Either yes or no. (Q13: “Do you perceive the word 'fatty' in 'fatty liver' as stigmatizing?”) Either yes or no. (Q14: “Do you perceive the word 'alcoholic' in 'non-alcoholic fatty liver' as stigmatizing?”) Either yes or no. This questionnaire was printed and filled out by the participants without physician interference or inclination, without the gathering of any identifying information. The analysis only included patients who completed the survey up to the last question. All respondents completed the survey anonymously. The study was done in compliance with the Declaration of Helsinki. All patient data were included in the study regardless of their answer, and the survey results were analyzed collectively and quantitatively.

### 2.4 Statistical Analysis

Data analyses were done using SPSS. Firstly, content validity was evaluated and reviewed by Hepatology experts mentioned in the panel. A pilot study was done to ensure construct validity, and internal consistency analyses were performed (Cronbach's alpha =0.7). Furthermore, a principal components analysis was performed to assess internal validity. Continuous data were expressed as Mean ± Standard deviation. For categorical data, the number of participants and percentages were calculated. The responses were tabulated as n (%).

## 3 RESULTS

### 3.1 Participant and Descriptive Data

The replies of 222 participants are presented in Table **1**. 87 (39.2%) were males with an average age of 46.56 ±12.06. Additionally, 117 (52.7%) had a high level of education, 110 (49.5%) belonged to the middle class based on their income level, and 143 (64.4%) resided in metropolitan regions.

### 3.2 Outcome Data

Stigma evaluation is presented in Table **2**. Only 61.7% (85/222) of participants have exhibited awareness of their existing liver disease. 92 individuals (41.4%) have never heard about NAFLD or NASH before. Additionally, 193 patients (86.9%) said they either had no knowledge of the distinction between NAFLD and NASH or had never heard of it. The healthcare provider seems to be the main source of knowledge about the disease among participants (43.7%).

Notably, in Fig. (**1**), a considerable proportion of respondents tended to trivialize the seriousness of the disease. When questioned about their level of worry if they were informed of having fatty liver disease on a scale of 1-5 (1= not at all concerned to 5= very concerned), only >43.3% of patients indicated that they would frequently or very concerned, 61.2%, 58.6%, 58.1% of these patients expressed a high level of worry if they were informed of having hepatitis C, hypertension or diabetes, respectively.

Additionally, in Table **2**, only 84 (37.8%) and 37 (16.7%) of patients thought that fatty liver disease could lead to cirrhosis or hepatocellular carcinoma, respectively. Similarly, for extra-hepatic manifestations, 82 (36.9%), 87 (39.2%), 52 (23.4%), and 34 (15.3%) of patients believed that there is any link between fatty liver and diabetes, cardiovascular disease, chronic kidney or extra-hepatic malignancies, respectively. Subsequently, 192 patients (86.5%) expressed their intention to decline a liver biopsy for the assessment of fatty liver, since they see the condition as insufficiently severe to warrant the procedure.

Concerning patients' perception of different “diagnostic terms,” presented in Fig. (**2**). A significant majority of 189 participants (85.1%) said that the inclusion of the phrase “alcoholic” in the name of a disease is associated with stigma. Conversely, 177 (79.7%) of participants said that they do not consider the term “fatty” to be stigmatizing.

Ultimately, in Table **2**, a total of 159 and 180 patients, accounting for 71.9% and 81.1% of the sample, expressed their lack of knowledge on how the disease can be diagnosed or treated, respectively. Nevertheless, 146 individuals (65.8%) of the participants expressed their ability to acquire knowledge regarding the potential impact of fatty liver disease on their well-being if they were informed of their susceptibility to it. Out of the total, 186 individuals (83.8%) expressed a preference for obtaining this information directly from their healthcare provider, such as their doctor or nurse.

## 4 DISCUSSION

Fatty liver disease, a fast-growing health issue that affects an estimated 30% of people worldwide, has become the primary cause of hepatocellular cancer and liver cirrhosis [13]. Although obesity, diabetes, and dyslipidemia are the most powerful risk factors of fatty liver disease, no drug has been approved as of yet [14, 15]. Few studies demonstrated the role of statins in fatty liver disease prevention, explained by statins' anti-inflammatory and anti-lipid properties [16].

Apart from our concern about raising awareness about fatty liver disease as a growing health concern linked to obesity, diabetes, and metabolic disorders. Fatty liver disease often progresses silently until it has progressed to an advanced stage. Early detection and lifestyle modifications can adversely affect disease progression. Additionally, educating people, including public health screenings and surveys, can help individuals understand risk factors, encourage proactive medical check-ups, and promote healthier habits. Despite the high prevalence of the disease, this research demonstrates a lack of awareness of its linked risk factors and complications. Besides the use of the term “alcohol” in the nomenclature of fatty liver disease linked to metabolic dysfunction is seen as stigmatizing, the term “fat” is not perceived as having a negative connotation. Furthermore, there is a lack of knowledge about the condition and a significant tendency among patients to trivialize the seriousness of the disease.

Despite the multiple positive attributes of the MAFLD term and its accompanied diagnostic criteria [11, 17], it was suggested recently that the term “fatty” is stigmatizing and requires a further shift to the largely adopted term MASLD, changing the “F” to “S” or “fatty” to “steatotic”. The current study failed to find any clues suggesting that this is the case. Consistently, another recent study from Mexico showed the same finding that while alcohol is stigmatizing, the word fatty was not perceived negatively [18]. Another global survey showed that the perception of NAFLD stigma varies significantly among patients, providers, geographic locations, and sub-specialties. Only 8% of patients perceived stigma compared to 38% of physicians [19]. This point has been precisely illustrated by liver patients’ spokespeople who clarified that to be stigmatizing must be visible, which does not apply to fatty liver, which is invisible. They have also highlighted that “in some cultures, being fat is regarded as a sign of good health [20].

Furthermore, it is worth noting that the terms “steatotic” and “fatty” have the same translation in the languages commonly spoken in our region, as well as in the majority of non-English languages. In addition, if individuals utilize the search engine 'Google' to inquire about the definition of “steatotic” or “steatosis”, they will find out that these terms refer to the build-up of fat in the liver. Likewise, doctors who struggle to convey the meaning of “steatotic” encounter obstacles beyond simply using the commonly used term “fatty”.

We believe cumulative results undermine the case for substituting “fatty” with “steatotic”. Ultimately, using a medical term like “steatotic” instead of a lay one as “fatty,” contradicts current efforts to enhance patient participation and empowerment, as medical terminology should aim to facilitate effective communication with patients [21, 22] These findings can be a sufficient basis to avoid any abrupt and unnecessary further changes in the nomenclature of fatty liver disease. Studies have shown that the transition to MAFLD has positive implications in increasing disease awareness among patients and healthcare providers [6, 7]. Using a completely new name instead of MAFLD will only undermine the momentum and stall progress, particularly with the observed very low awareness level among patients with fatty liver disease.

## CONCLUSION

This study demonstrates that whereas the term “alcohol” in the nomenclature for fatty liver illness linked to metabolic dysfunction is seen as stigmatizing, surprisingly, the word “fat” is not regarded in a negative sense. And the claim of stigmatization does not exist. Importantly, there seems to be low awareness of the disease, and patients tend to trivialize its seriousness. The key aspect is striking to maintain the balance between disease-related stigma, patient motivation, and clarity of terminology. The path ahead is to redirect the energy now focused on intensifying the feelings of uncertainty and dread caused by the transition, and instead capitalize on the momentum created by the introduction of MAFLD. Our study had limitations. We suggest a larger sample size, inclusion of control groups, and standardized measurement tools to minimize potential bias (selection, sample size, observer, and reporting bias). Also, the proposed survey was adopted in our locality in different institutions, and we suggest that it be applied in other counties to disseminate our results.

## Figures and Tables

**Fig. (1):**
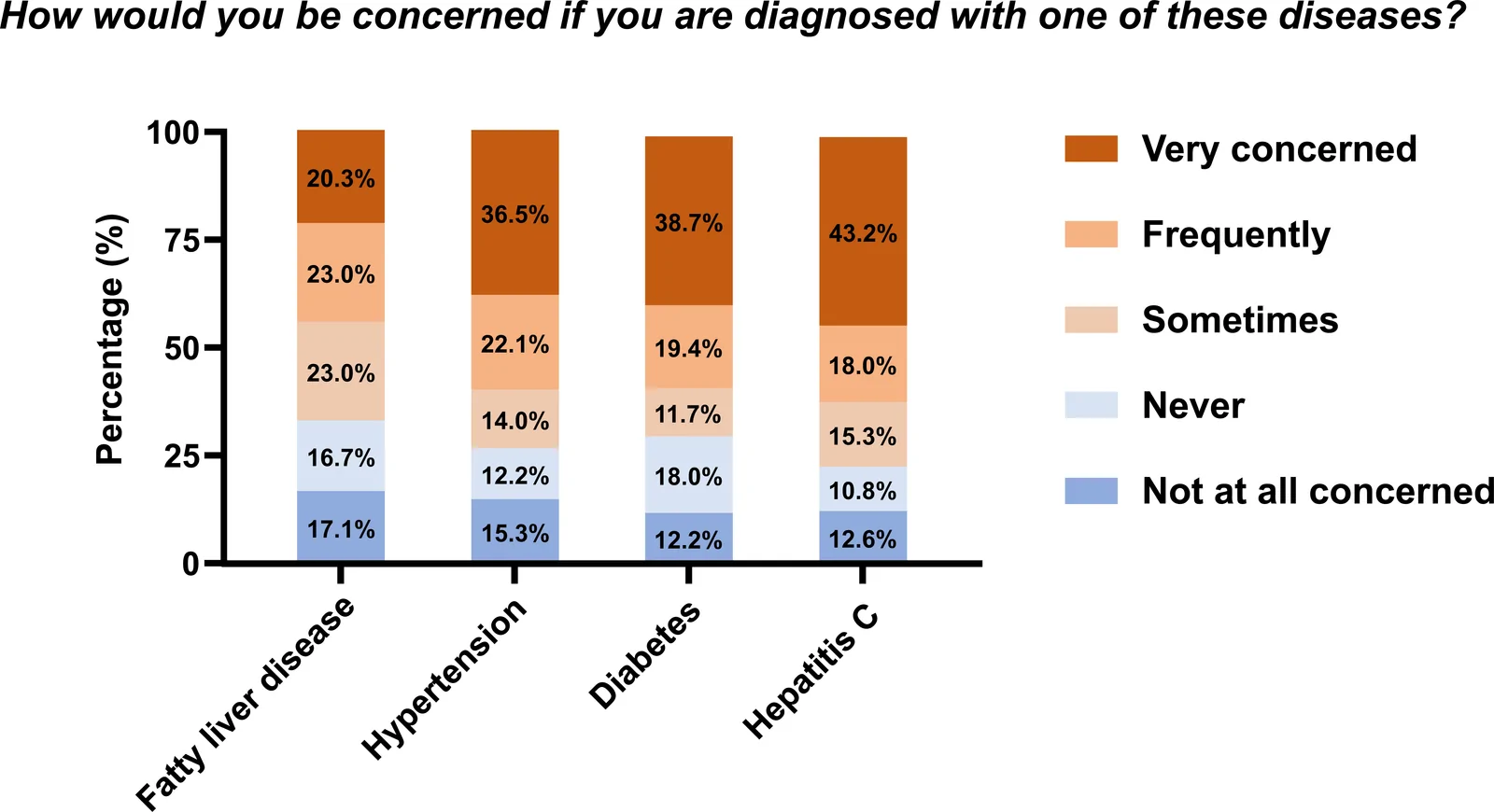
Patients’ self-reported perception of the concern about the seriousness of fatty liver disease compared to other common diseases.

**Fig. (2):**
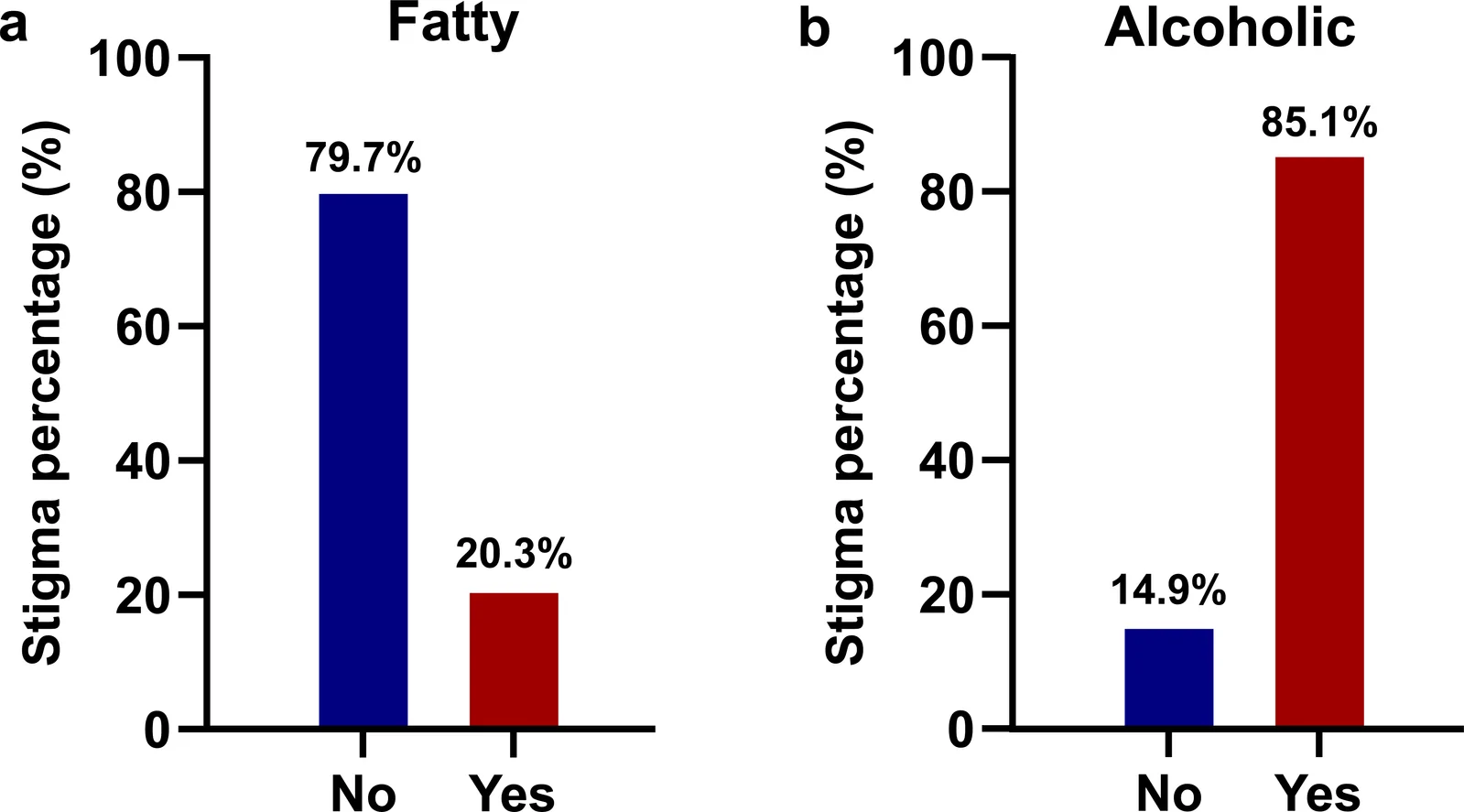
Patients’ self-reported discomfort with the diagnostic terms for fatty liver disease.

**Table 1:** Characteristics of subjects with fatty liver disease.

**Characteristics**	Fatty Liver Disease **(n = 222)**	
	**Mean / Number**	**Range** **(min-max) / %**
Age (year)	46.56 ±12.06	19-81
Male (%)	87	39.2%
High-level education (%)	117	52.7%
High income (%)	110	49.5%
City resident (%)	143	64.4%

**Abreviations:** min: minimum, max: maximum.

**Table 2:** Patients’ self-reported history of stigma or discrimination.

**Question**	**NASH (222) N (%)**
Awareness of the existing liver disease	-
No	85 (38.3%)
Yes	137 (61.7%)
Heard of NASH/NAFLD before	-
No	92 (41.4%)
Yes	129 (58.1%)
Source of awareness	-
From a healthcare provider	97 (43.7%)
From a friend or a family member	14 (6.3%)
From the news or media	12 (5.4%)
From the Internet	10 (4.5%)
From other sources	0 (0.0%)
Non	89 (40.1%)
Heard about Liver Cirrhosis	-
No	93 (41.9%)
Yes	129 (58.1%)
If know the difference between NAFLD and NASH?	-
No	193 (86.9%)
Yes	29 (13.1%)
If know that Fatty liver can cause	-
Cirrhosis	-
No	138 (62.2%)
Yes	84 (37.8%)
Hepatocellular carcinoma	-
No	185 (83.3%)
Yes	37 (16.7%)
Diabetes	-
No	140 (63.1%)
Yes	82 (36.9%)
CVD	-
No	135 (60.8%)
Yes	87 (39.2%)
CKD	-
No	170 (76.6%)
Yes	52 (23.4%)
Cancers (non-hepatic)	-
No	188 (84.7%)
Yes	34 (15.3%)
Learn affection of NAFLD on health	-
No	76 (34.2%)
Yes	146 (65.8%)
How to receive the information:	-
a. Directly from a doctor or nurse	186 (83.8%)
b. Written materials	22 (9.9%)
c. Reordered materials	13 (5.9%)
Diagnosis idea	-
No	159 (71.9%)
Yes	62 (28.1%)
Accept biopsy	-
No	192 (86.5%)
Yes	30 (13.5%)
Treatment idea	-
No	180 (81.1%)
Yes	42 (18.9%)

**Abbreviations:** NASH: non-alcoholic steatohepatitis, NAFLD: non-alcoholic fatty liver disease, CKD: chronic kidney disease, CVD: chronic cardiovascular disease.

## Data Availability

All data generated or analyzed during this study are included in this published article.

## LIST OF ABBREVIATIONS

MAFLD: Metabolic Dysfunction-Associated fatty Liver Disease
MASLD: Metabolic Dysfunction-Associated Steatotic Liver Disease

## STANDARDS OF REPORTING

STROBE guidelines were followed.

## References

[1] Chan K.E., Koh T.J.L., Tang A.S.P. (2022). Global prevalence and clinical characteristics of metabolic associated fatty liver disease. A Meta-analysis and systematic review of 10,739,607 individuals.. J. Clin. Endocrinol. Metab..

[2] Eslam M., El-Serag H.B., Francque S., Sarin S.K., Wei L., Bugianesi E., George J. (2022). Metabolic (dysfunction)-associated fatty liver disease in individuals of normal weight.. Nat. Rev. Gastroenterol. Hepatol..

[3] Sarin S.K., Kumar M., Eslam M., George J., Al Mahtab M., Akbar S.M.F., Jia J., Tian Q., Aggarwal R., Muljono D.H., Omata M., Ooka Y., Han K.H., Lee H.W., Jafri W., Butt A.S., Chong C.H., Lim S.G., Pwu R.F., Chen D.S. (2020). Liver diseases in the Asia-Pacific region: a Lancet gastroenterology & hepatology commission.. Lancet Gastroenterol. Hepatol..

[4] Li X., Hu C., Luo S., Dai F., Li C., Zhou W., Wang J., Chen H., Wang Z., Long T., Jiang L., Tang C. (2024). Cav3.2 deletion attenuates nonalcoholic fatty liver disease in mice.. Gene.

[5] Alem S.A., Gaber Y., Abdalla M., Said E., Fouad Y. (2021). Capturing patient experience: A qualitative study of change from NAFLD to MAFLD real-time feedback.. J. Hepatol..

[6] Fouad Y., Gomaa A., Semida N., Ghany W.A., Attia D. (2021). Change from NAFLD to MAFLD increases the awareness of fatty liver disease in primary care physicians and specialists.. J. Hepatol..

[7] Fouad Y., Abdel Salam S., AbdAllah M., Attia D. (2022). The change from NAFLD to MAFLD expands fatty liver information flow.. Hepatol. Res..

[8] Eslam M., Newsome P.N., Sarin S.K., Anstee Q.M., Targher G., Romero-Gomez M., Zelber-Sagi S., Wai-Sun Wong V., Dufour J.F., Schattenberg J.M., Kawaguchi T., Arrese M., Valenti L., Shiha G., Tiribelli C., Yki-Järvinen H., Fan J.G., Grønbæk H., Yilmaz Y., Cortez-Pinto H., Oliveira C.P., Bedossa P., Adams L.A., Zheng M.H., Fouad Y., Chan W.K., Mendez-Sanchez N., Ahn S.H., Castera L., Bugianesi E., Ratziu V., George J. (2020). A new definition for metabolic dysfunction-associated fatty liver disease: An international expert consensus statement.. J. Hepatol..

[9] Eslam M., Sanyal A.J., George J., Sanyal A., Neuschwander-Tetri B., Tiribelli C., Kleiner D.E., Brunt E., Bugianesi E., Yki-Järvinen H., Grønbæk H., Cortez-Pinto H., George J., Fan J., Valenti L., Abdelmalek M., Romero-Gomez M., Rinella M., Arrese M., Eslam M., Bedossa P., Newsome P.N., Anstee Q.M., Jalan R., Bataller R., Loomba R., Sookoian S., Sarin S.K., Harrison S., Kawaguchi T., Wong V.W-S., Ratziu V., Yilmaz Y., Younossi Z., International Consensus Panel (2020). MAFLD: A consensus-driven proposed nomenclature for metabolic associated fatty liver disease.. Gastroenterology.

[10] Eslam M., Alkhouri N., Vajro P., Baumann U., Weiss R., Socha P., Marcus C., Lee W.S., Kelly D., Porta G., El-Guindi M.A., Alisi A., Mann J.P., Mouane N., Baur L.A., Dhawan A., George J. (2021). Defining paediatric metabolic (dysfunction)-associated fatty liver disease: An international expert consensus statement.. Lancet Gastroenterol. Hepatol..

[11] Alharthi J., Gastaldelli A., Cua I.H., Ghazinian H., Eslam M. (2022). Metabolic dysfunction-associated fatty liver disease: A year in review.. Curr. Opin. Gastroenterol..

[12] Rinella M.E., Lazarus J.V., Ratziu V., Francque S.M., Sanyal A.J., Kanwal F., Romero D., Abdelmalek M.F., Anstee Q.M., Arab J.P., Arrese M., Bataller R., Beuers U., Boursier J., Bugianesi E., Byrne C.D., Narro G.E.C., Chowdhury A., Cortez-Pinto H., Cryer D.R., Cusi K., El-Kassas M., Klein S., Eskridge W., Fan J., Gawrieh S., Guy C.D., Harrison S.A., Kim S.U., Koot B.G., Korenjak M., Kowdley K.V., Lacaille F., Loomba R., Mitchell-Thain R., Morgan T.R., Powell E.E., Roden M., Romero-Gómez M., Silva M., Singh S.P., Sookoian S.C., Spearman C.W., Tiniakos D., Valenti L., Vos M.B., Wong V.W.S., Xanthakos S., Yilmaz Y., Younossi Z., Hobbs A., Villota-Rivas M., Newsome P.N., NAFLD Nomenclature consensus group (2024). A multisociety Delphi consensus statement on new fatty liver disease nomenclature.. Ann. Hepatol..

[13] Darci-Maher N., Alvarez M., Arasu U.T., Selvarajan I., Lee S.H.T., Pan D.Z., Miao Z., Das S.S., Kaminska D., Örd T., Benhammou J.N., Wabitsch M., Pisegna J.R., Männistö V., Pietiläinen K.H., Laakso M., Sinsheimer J.S., Kaikkonen M.U., Pihlajamäki J., Pajukanta P. (2023). Cross-tissue omics analysis discovers ten adipose genes encoding secreted proteins in obesity-related non-alcoholic fatty liver disease.. EBioMedicine.

[14] Ying Z., van Eenige R., Ge X., van Marwijk C., Lambooij J.M., Guigas B., Giera M., de Boer J.F., Coskun T., Qu H., Wang Y., Boon M.R., Rensen P.C.N., Kooijman S. (2023). Combined GIP receptor and GLP1 receptor agonism attenuates NAFLD in male APOE-3-Leiden.CETP mice.. EBioMedicine.

[15] Li Z., Zhang B., Liu Q., Tao Z., Ding L., Guo B., Zhang E., Zhang H., Meng Z., Guo S., Chen Y., Peng J., Li J., Wang C., Huang Y., Xu H., Wu Y. (2023). Genetic association of lipids and lipid-lowering drug target genes with non-alcoholic fatty liver disease.. EBioMedicine.

[16] Ayada I., van Kleef L.A., Zhang H., Liu K., Li P., Abozaid Y.J., Lavrijsen M., Janssen H.L.A., van der Laan L.J.W., Ghanbari M., Peppelenbosch M.P., Zheng M.H., de Knegt R.J., Pan Q. (2023). Dissecting the multifaceted impact of statin use on fatty liver disease: A multidimensional study.. EBioMedicine.

[17] Kawaguchi T., Tsutsumi T., Nakano D., Eslam M., George J., Torimura T. (2022). MAFLD enhances clinical practice for liver disease in the Asia-Pacific region.. Clin. Mol. Hepatol..

[18] Ramírez-Mejía M.M., Qi X., Abenavoli L., Méndez-Sánchez  N. ( 2023). The myth of the stigma of fatty liver: What does the evidence show.. Ann. Hepatol..

[19] Méndez-Sánchez N., Pal S.C., Fassio E., Díaz-Ferrer J., Prado-Robles J.A. (2023). MAFLD: Perceived stigma: A single-center mexican patient survey.. Hepatol. Int..

[20] Younossi Z.M., Alqahtani S.A., Alswat K., Yilmaz Y., Keklikkiran C., Funuyet-Salas J., Romero-Gómez M., Fan J.G., Zheng M.H., El-Kassas M., Castera L., Liu C.J., Wai-Sun Wong V., Zelber-Sagi S., Allen A.M., Lam B., Treeprasertsuk S., Hameed S., Takahashi H., Kawaguchi T., Schattenberg J.M., Duseja A., Newsome P.N., Francque S., Spearman C.W., Castellanos Fernández M.I., Burra P., Roberts S.K., Chan W.K., Arrese M., Silva M., Rinella M., Singal A.K., Gordon S., Fuchs M., Alkhouri N., Cusi K., Loomba R., Ranagan J., Eskridge W., Kautz A., Ong J.P., Kugelmas M., Eguchi Y., Diago M., Yu M.L., Gerber L., Fornaresio L., Nader F., Henry L., Racila A., Golabi P., Stepanova M., Carrieri P., Lazarus J.V., Global NASH Council (2024). Global survey of stigma among physicians and patients with nonalcoholic fatty liver disease.. J. Hepatol..

[21] Shiha G., Korenjak M., Casanovas T., Mooney V., Sigurðardóttir S., Koulla Y., Soliman R. (2022). MAFLD 2022: An ELPA/ALPA/EASO-ECPO joint statement on disease stigma.. J. Hepatol..

[22] Shiha G., Korenjak M., Eskridge W., Casanovas T., Velez-Moller P., Högström S., Richardson B., Munoz C., Sigurðardóttir S., Coulibaly A., Milan M., Bautista F., Leung N.W.Y., Mooney V., Obekpa S., Bech E., Polavarapu N., Hamed A.E., Radiani T., Purwanto E., Bright B., Ali M., Dovia C.K., McColaugh L., Koulla Y., Dufour J.F., Soliman R., Eslam M. (2021). Redefining fatty liver disease: An international patient perspective.. Lancet Gastroenterol. Hepatol..

